# Classification of Indoor Human Fall Events Using Deep Learning

**DOI:** 10.3390/e23030328

**Published:** 2021-03-10

**Authors:** Arifa Sultana, Kaushik Deb, Pranab Kumar Dhar, Takeshi Koshiba

**Affiliations:** 1 Department of Computer Science and Engineering, Chittagong University of Engineering & Technology (CUET), Chattogram 4349, Bangladesh; arifa.z@eastdelta.edu.bd (A.S.); pranabdhar81@cuet.ac.bd (P.K.D.); 2 Faculty of Education and Integrated Arts and Sciences, Waseda University, 1-6-1 Nishiwaseda, Shinjuku-ku, Tokyo 169-8050, Japan; tkoshiba@waseda.jp

**Keywords:** human fall classification, deep learning, recurrent neural network (RNN), convolutional neural network (CNN), gated recurrent unit (GRU)

## Abstract

Human fall identification can play a significant role in generating sensor based alarm systems, assisting physical therapists not only to reduce after fall effects but also to save human lives. Usually, elderly people suffer from various kinds of diseases and fall action is a very frequently occurring circumstance at this time for them. In this regard, this paper represents an architecture to classify fall events from others indoor natural activities of human beings. Video frame generator is applied to extract frame from video clips. Initially, a two dimensional convolutional neural network (2DCNN) model is proposed to extract features from video frames. Afterward, gated recurrent unit (GRU) network finds the temporal dependency of human movement. Binary cross-entropy loss function is calculated to update the attributes of the network like weights, learning rate to minimize the losses. Finally, sigmoid classifier is used for binary classification to detect human fall events. Experimental result shows that the proposed model obtains an accuracy of 99%, which outperforms other state-of-the-art models.

## 1. Introduction

Human fall detection system is an important segment of assistive technology since living assistances are very much obligatory for many people. There has been a remarkable emersion in the elderly population in Bangladesh as well as in western countries over recent years. The statistics on human fall detection have exposed that falls play a key role in injurious death for elders more than 79 years of age [1]. According to the review from the National Institutes of Health of the United States, approximately 1.6 million aged people are sustaining fall-related excoriations in the U.S. every year [2]. Meanwhile, China is facing the fastest aging population in human history, the population will rise to about 35% by 2050 [2] from 2020. A study shows that about 93% of the elders among which 29% live alone in a house [3]. It was verified that about 50% of the aged people lying on the floor because of fall events for more than one hour, will die within six months even though they do not have any direct injuries [4].

Another statistic provided by the Public Health Agency of Canada [5] reported mentionable data. In 2026, one Canadian older than 65 will be out of five where in 2001 the portion was eight to one. It is notable that 93% of elderly people stay in their private house among which 29% of them lead a lonely life [5]. Again almost 62% of injury-related hospitalizations for elders are the result of falls [6].

To identify fall at right, in recent years various methods are proposed using advanced devices like wearable sensors, accelerometers, gyroscope, magnetometers and so on. However, this is not an effective solution since it is impractical to wear a device for a long time [7].

Therefore, it is indispensable to initiate a penetrating surveillance system for senior people, which can immediately and automatically detect fall actions inside the room and notify the state to the caretakers. This is only possible when a sensor-based alarm generation system is placed in the living room.

In this paper, a deep learning and vision-based framework is proposed for human fall detection and classification that can also monitor old people in the indoor environment incorporating a convolutional neural network (CNN) with the recurrent neural network (RNN). Among different types of RNN, gated recurrent unit is integrated along with CNN. We have also explored some transfer learning models like VGG16, VGG19 followed by GRU to classify human fall action. Besides, we have also assessed our proposed model using the two most prominent datasets, UR fall detection dataset and Multiple cameras fall dataset. Our proposed model shows an impressive performance using these datasets compared to other existing models.

Moreover, with the expansion of technology, human beings are leading a sedentary lifestyle which has numerous negative impacts [8]. Because of this lifestyle, people are suffering from many more diseases like chronic health disease, muscle weakness, labyrinthitis, osteoporosis. Besides, the number of lonely living beings is increasing with the advancement of technology which represents the necessity of a monitoring system to detect adverse events [9]. This model will be applicable in the medical alert system and even in the old home for monitoring individuals. However, the key contributions of our proposed model are enlisted below:

- Building a scratch model combining CNN with GRU to classify human fall events from daily living activities.
- Exploring a transfer learning approach through training some recent pre-trained models like Xception, VGG integrating with GRU and evaluating their performance.
- Fine-tuning two pre-trained models: VGG16 and VGG19 along with GRU.
- Assessing the performance of the proposed model with some deep learning models like 3DCNN, 1DCNN incorporating with GRU.
- Executing some best performing recurrent neural networks: Long and Short Term Memory (LSTM), Bidirectional LSTM (BI-LSTM) combining with the proposed 2DCNN network and evaluating performance over two challenging datasets, UR fall detection dataset and Multiple cameras fall dataset.

The later part of this paper is well organized as follows—Section 2 represents the literature discussion on human fall events, Section 3 describes the workflow of the proposed architecture, Section 4 presents experimental details to evaluate the efficiency of the proposed model and Section 5 discusses the limitation of the proposed model along with potential future work.

## 2. Related Work

In this decade, among different types of deep learning models [10], convolutional neural network (CNN) has acquired immense success in computing like image segmentation [11], object recognition [12], natural language processing [13], image understanding [14] and machine translation [15], which requires a huge training dataset to complete the set up.

A study by Stone, Erik E. and Marjorie S. reveals that falls of older people at home happens most of the time in dark conditions [16]. Sowmya K. and Kang-Hyun Jo [17] classify fall events in a cluttered indoor environment by lessening occlusion effects. However, the knowledge of a series of poses is a key to detect non-fall from fall. For foreground extraction, a frame differencing method is implemented and the human silhouette is extracted using an ellipse fit. Binary support vector machine (SVM) is applied to differentiate fall frames from the non-fall. But SVM underperforms in case of an insufficient training data sample.

Convolutional neural network (CNN) is used to identify different poses in [18]. Here, in different illumination states, the background subtraction method misclassifies some datasets because of shadow. As a result, it generates false predictions in bending, crawling and sitting positions. Tamura et al. [19] developed a human fall detection system using a gyroscope and an accelerometer. When a fall action is detected it triggers a wearable airbag. To design the system, 16 subjects have been produced to identify mimicked falls and a thresholding technique is applied to perform this action.

A real-life action recognition system is overviewed in [20] using deep bidirectional LSTM (DB-LSTM) and convolutional neural network (CNN). Here, DB-LSTM recognizes hidden sequential patterns in the features where CNN extracts data from video frames. But it shows false projection at the identical background and occluded environment.

Du et al. [21] conducted research on convolutional neural network (CNN) to extract the skeleton joint map from different images. However, the result can be developed if the recurrent neural network (RNN) is implemented properly as in [22]. Anderson et al. [23] procreated a surveillance environment using multiple cameras. Human silhouettes captured from the cameras are converted to 3-D representations known as voxel person. Finally, a fall event is classified from linguistic summarizations of temporal fuzzy inference curves, which represent the states of a voxel person.

Different types of human action can be represented as the movement of the skeleton as the human body is an articulated system. The 2D skeleton is extracted from RGB sequences in [24] using the deeper-cut method and long short term memory (LSTM) is implemented to identify five several actions. However, It is more challenging on processing speed and recognition performance.

The faster R-CNN method is applied in [25], which achieves an accuracy of 95.5% as it cannot properly classify fall events when a person is sitting on a sofa or a chair. A PCANet model is trained followed by SVM classifier in [26], which obtains less sensitivity of 88.87% as it cannot identify fall events properly. Moreover, SVM underperforms as fine-tuning hyperparameters in SVM is not so easy.

In [27] curvature scale space (CSS) features are extracted from human silhouettes and an extreme learning machine (ELM) classifier is used to identify fall action. However, this experiment achieves 86.83% accuracy as it misclassifies the lying and walking position of humans as the silhouette of a falling and a lying person is similar.

A two-stage human fall classification model is proposed in [28], which achieves an accuracy of 97.34%. To identify human posture from the human skeleton, at the preprocessing stage it considers deflection angles along with spine ratio. To extract the human skeleton it uses OpenPose and to identify confusing daily living actions from fall events, a time-continuous recognition algorithm is developed. However, this model misclassifies workout motions and for increasing accuracy, it has proposed to develop a deep learning model in the future.

Besides, Chen et al. [29] detect human fall events from the human skeleton information by OpenPose and obtains an accuracy of 97%. To identify fall action three efficient parameters are considered here like centerline angle between human body and ground, speed of descent at the center point of the hip joint, the ratio of width and height of external rectangle surrounding the human body. However, this model is unable to classify partially occluded human actions.

Moreover, A vision-based human fall detection model is proposed by Chen et al. [7] in the case of complex background. They perform the mask R-CNN method to extract the object from a noisy background. Afterward, for fall action detection, attention-guided Bi-directional LSTM is applied and it acquires 96.7% accuracy. However, this model cannot identify the behavior of multiple people living in the same room.

Nowadays, different types of wearable sensors, that is, accelerometers, buttons are most frequently used to detect falls at the right time. However, using such detectors is uncomfortable and most of the time, older people forget to wear these sensors. Moreover, using a help button is worthless if the person has fainted or is immobilized. Such a framework for elderly fall classification and notification is proposed in [9]. Here, the tri-axial acceleration of human movement is measured with a cell phone. Both the time domain and frequency domain features are considered here. After feature extraction and pre-processing they have performed a deep belief network with a view to training and testing the system. It shows an accuracy of 97.56% sensitivity along with 97.03% specificity. However, for elder people, it is not always possible to carry a cell phone in an indoor environment.

In [8], the authors conducted a highly promising experiment to develop a hybrid model using a machine learning method combining with deep learning. After analyzing different comparative experiments, they proposed an architecture of CNN with LSTM for posture detection with an accuracy of 98%. This CNN model has been designed with 10 layers without any batch normalization. Unnormalized data and less dropout rate of 20% can lead to a huge training time of the dataset and the performance of the model may also be affected.

Table 1 represents the summarization of this literature discussion.

As deep learning models have outperformed state-of-the-art models, there are many scopes for innovation and development in this research area. However, a computer vision-based model is proposed to classify fall events at the right time, which provides whole information regarding the movement of a person.

## 3. Workflow of Proposed Architecture

**Figure 1:**
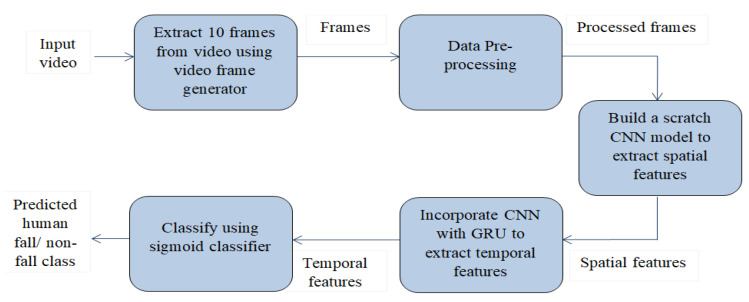
Workflow of the proposed human fall classification model.

Elderly people monitoring is done through a digital video camera, which will be placed in the room as there are some distance limitations in the Kinect camera. Although it has some privacy concerns, it will give us information about surroundings in case of fall events. Sequential frames are generated from videos of variable length. Frames are passed into the convolutional neural network to extract key features. After that these features are passed into a gated recurrent unit. The output from GRU is passed to a sigmoid classifier to predict the class. Figure 1 illustrates an overview of the proposed network.

### 3.1. Frame Generation from Video

There are approximately 300 videos from different datasets of various durations. From each video, we need to pass a sequence of frames to CNN. As mentioned earlier, we picked 10 distributed images from the whole video rather than considering all other frames. The algorithm to generate frame sequence from video is given below. We performed a frame differencing method in Algorithm 1 along with other operations for tweaking important and informative frames from the video.
**Algorithm 1** Video to frame generation.Input: Number of frames need to extractOutput: Batches of imagesmove_detect = Statistical mean threshold valueIf move_detect >  0f = frames [0]f = convert image from RGB to GRAYlast = fimportant_frame = []for frame number i = 1 to length(frames)f = frame[i]cp = convert image from RGB to GRAYdelta = absolute difference(cp, last)threshold = threshold ( delta, thresholdvalue, 255, threshold_binary)threshold = dialate (threshold, structure element, iteration)if np.mean(threshold) >  move_detectmark as important_frameendlast = cpendnb_ignore = length (important_frame)/nb_frameif length(important_frame) >  nb_framecollect frame from last and ignore nb_ignoreendend

### 3.2. Preprocessing

For improving image properties, eliminating noisy artifacts and enhancing certain features, it is necessary to preprocess data. We have performed preprocessing by three steps—resizing, augmentation and normalization. In order to minimize computational cost frames are resized to 150 × 150.

On the resized image, augmentation is performed, which transforms frames at each epoch of training. For augmentation, we have performed zoom, horizontal flip, rotation, width shift and height shift. This helps for better generalization of this model.

### 3.3. Convolutional Neural Network

Convolutional neural network is a type of deep neural network which extracts key features from images using learnable weights and biases and can differentiate one object from another. Comparing with other classification algorithms, it requires much lower preprocessing of images. It consists of an input layer, an output layer along with a series of hidden layers. The hidden layers typically consist of a stack of convolutional layers which perform pixel-wise convolution or multiplication operation and generate a convolved image. The activation function used here is a rectified linear unit (ReLU) layer which is followed by a series of pooling layers and fully connected layers.

There are three types of the pooling operation. These are max pooling, min pooling, and average pooling operation. Among these, the max-pooling operation is most frequently used as it minimizes computational cost along with learnable parameters.

For extracting features from images, CNN uses this series of convolutional layers followed by different pooling layers, flatten layer as well as fully connected layer. Each layer works with different activation functions. Following this, the proposed architecture is also designed with a series of convolution layers with ‘ReLU’ activation function. The ReLU activation function generates a rectified feature map as output. It does not excite all neurons at a time. When the output of linear transformation becomes zero, the neurons will be discarded. Although there are different types of pooling layers, we have used the max-pooling layer

### 3.4. Custom 2DCNN-GRU Architecture

**Figure 2:**
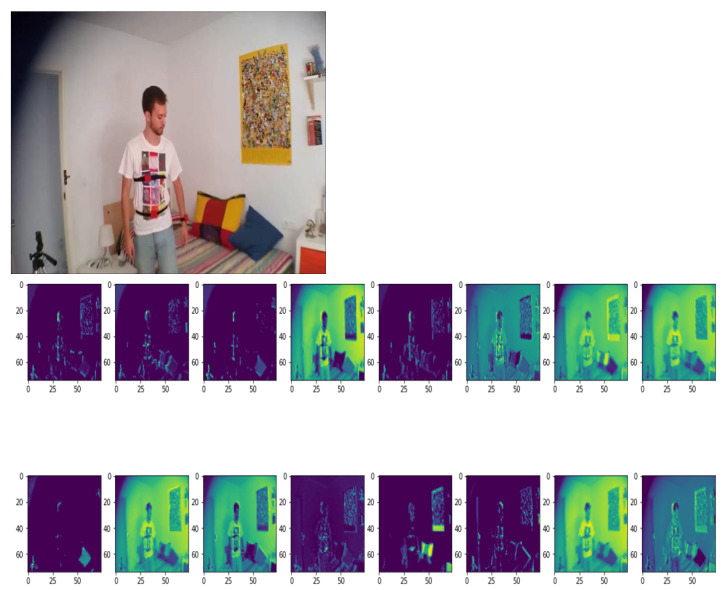
Visualization of the output of convolution layer for human fall classification.

**Figure 3:**
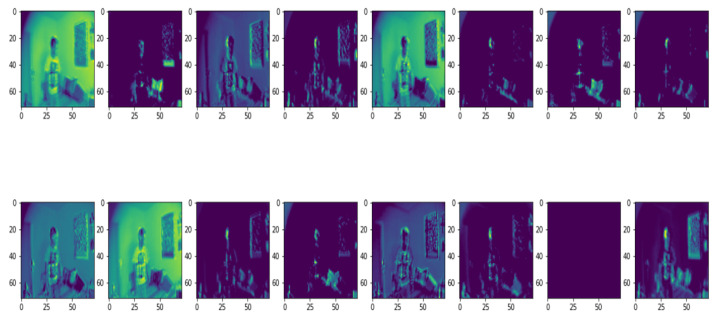
Visualization of the output of max-pooling layer for human fall classification.

Preprocessed data are fed to CNN. We have proposed a CNN architecture for extracting spatial features. As the environment in the dataset is less complex, 16, 32, 64, 128, 256, and 512 kernels are used sequentially in the convolution layer to extract features from the image. Weights are tuned for each layer depending on the activation function. This network gives much more accuracy than other pre-trained networks of CNN because an experiment is done for choosing kernel initializer along with activation function. In this model he_uniform kernel initializer is used for choosing the initial kernel and updating weights for training data with ReLU activation function. We have also conducted an experiment for choosing appropriate momentum for batch normalization. We have performed batch normalization with a momentum of 0.9. Batch normalization standardizes the mean and variance to make learning stable. Here, it takes 83 s to complete an epoch using batch normalization. Without batch normalization, it takes 99 s for an epoch in case of training the dataset. After batch normalization, the max pooling operation with stride (2, 2) is performed to extract the strong edges in the image. This also helps to remove shallow edges corresponding to noise. The output of the convolution layer and max-pooling layer are shown, respectively, in Figure 2 and Figure 3. Figure 2 depicts the convolved image and Figure 3 illustrates the pooled feature map.

**Figure 4:**
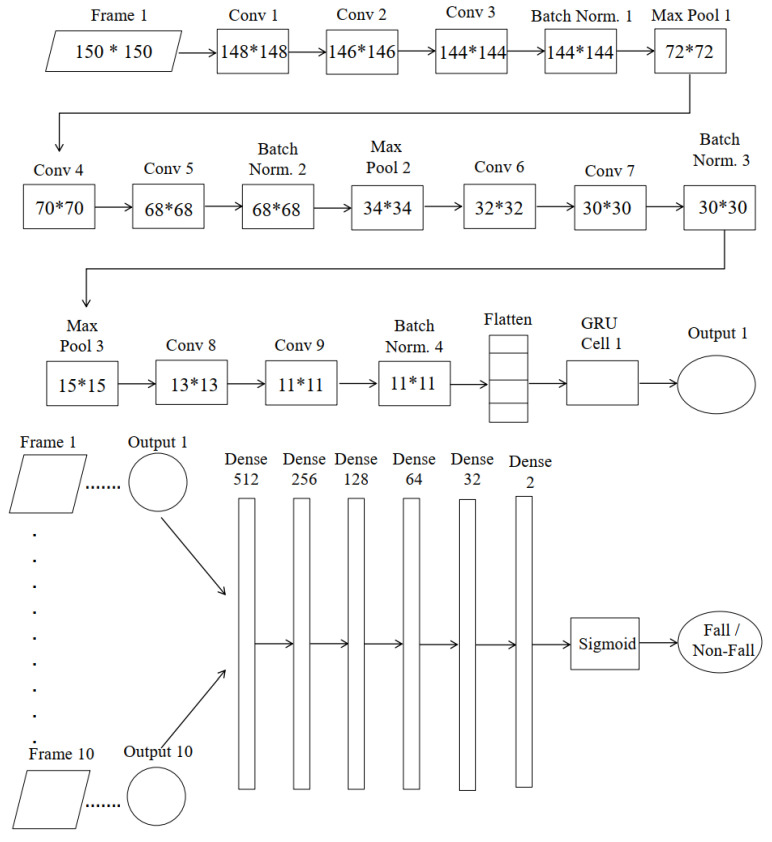
Proposed 2DCNN-GRU architecture to classify human fall.

After that, a time-distributed layer is used with 512 nodes to prepare data for RNN. Then GRU cells are used to follow the temporal dependency of video frames. The output of the GRU cell is passed to the dense layer. For eliminating overfitting, we have experimented on the dropout rate which is described in this paper. In this regard, a dropout rate of 0.5 is used in the dense layer for better accuracy. Adam optimizer with a learning rate of 0.0001 is used in the model. The detail of the proposed 2DCNN-GRU architecture is depicted in Figure 4.

### 3.5. Gated Recurrent Unit (GRU)

Among all kinds of human movements, to classify fall events, we need to consider temporal features along with spatial features. The recurrent neural network can extract temporal features by remembering necessary information from the past. However, during this operation, it faces vanishing and exploding gradient problems. In our model, we have used the gated recurrent unit (GRU) network to solve this problem of RNN using an update gate and a reset gate, which are the vectors to decide what information needs to be passed as output. These gates can be trained to hold information from long ago, without erasing new input through time but pass relevant information for prediction to the next time steps. GRU gives a much better result than a long short term memory (LSTM) cell because of its simple architecture.

**Figure 5:**
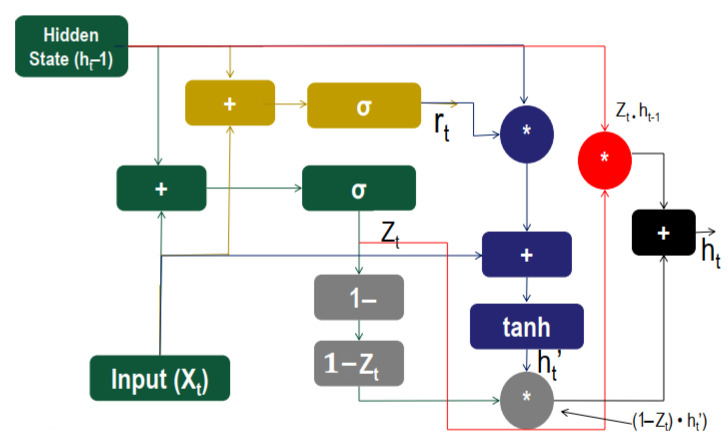
Gated Recurrent Unit (GRU) Cell.

Figure 5 depicts the architecture of a GRU cell. There are three gates in GRU called the Update gate, the Reset gate, and the Current memory gate, which has no internal cell state. The Update gate identifies significant preceding information from the antecedent time steps, which is required to be passed along with future information. The reset gate decides how much of the past information needs to be forgotten. The current state gate determines the current state information along with the relevant information from the past.

Mathematical equations learned from [30] are given below which are used to perform these operations:

(1) $$Update\;gate,Z_{t}=\sigma \left(W^{\left(Z\right)}x_{t}+U^{\left(z\right)}h_{\left(t-1\right)}\right)$$

(2) $$Reset\;gate,r_{t}=\sigma \left(W^{\left(r\right)}x_{t}+U^{\left(r\right)}h_{\left(t-1\right)}\right)$$

(3) $$Current\;memory\;gate,{\hat{h}}_{t}=\tanh\left(Wx_{t}+r_{t}*Uh_{\left(t-1\right)}\right)$$

(4) $$Final\;memory,h_{t}=Z_{t}*h_{\left(t-1\right)}+\left(\left(1-Z_{t}\right)*{\hat{h}}_{t}\right).$$

Here, $x_{t}$ represents the mini batch input, $h_{\left(t-1\right)}$ acts as the hidden state of utmost time step, $W^{\left(z\right)}$ and $W^{\left(r\right)}$ are current weight parameters and $U^{\left(z\right)}$, $U^{\left(r\right)}$ are updated weight parameters.

The output of GRU cell is passed to the dense layer with a dropout rate of 50%. Finally, sigmoid activation function [31] is applied for binary classification of fall and non-fall action through the following equations.

(5) $$Sigmoid\;activation\;function,S\left(x\right)=e^{x}/\left(e^{x}+1\right).$$

Deep learning models are efficiently used to lessen uncertainty and entropy measures the uncertainty level. Binary cross entropy plays a significant role to alleviates incongruity between the explorative distribution of training data and the distribution incited by the model. Here following binary cross-entropy loss function [32] equation is used to compare the predicted class with the actual class.

(6) $$Binary\;cross\;entropy\;loss,L=-\sum_{i=1}^{2}t_{i}log\left(p_{i}\right).$$

Here, $t_{i}$ denotes the truth value and $p_{i}$ represents the sigmoid probability of *i*th class.

## 4. Experiments

For executing this experiment, we have used the machine of a configuration of AMD Ryzen 7 2700X Eight-core 3.7 GHz Processor, 32 GB RAM, NVIDIA GEFORCE RTX 2060 SUPER of 8 GB GPU memory. We have also used Google colab for faster execution.

### 4.1. Dataset Description

We have conducted this experiment on the UR fall detection dataset and multiple cameras fall dataset to examine the accuracy of our proposed model.

#### 4.1.1. UR Fall Detection Dataset

UR fall detection dataset is one of the most benchmark datasets which comprises 70 indoor videos among which 30 fall events and 40 daily living activities. Here, fall activities are recorded using two Kinect cameras whereas daily living activities are recorded using only one camera. In this dataset, there are 30 frames per video. Each video possesses a resolution of 640 × 240.

#### 4.1.2. Multiple Cameras Fall Dataset

Multiple cameras fall dataset is one of the most extensive datasets which is widely used to classify human fall action. It includes 192 videos where 96 videos represent fall events and 96 videos are of regular indoor activities. This dataset is recorded with 24 scenarios which represent 9 different activities like walking, falling, lying on the ground, crouching, and so on. Eight different cameras are used to capture each activity. Each video has a frame rate of 30 fps with a frame size of 720 × 480.

### 4.2. Evaluation Metrices

The proposed model is also evaluated in terms of accuracy, precision, sensitivity, specificity and F1-score. Accuracy demonstrate the rate of correctly classified data using following equation [33]:

(7) Accuracy=(TP+TN)/(TP+TN+FP+FN),

where *TP* represents the true positive rate, that is, the detected result is a fall, which is actually a fall event, *TN* represents the true negative rate, that is, the detected event is a non-fall, which is actually a non-fall event, FP represents the false positive rate, that is, the detected event is a fall, which is a non-fall event in real-time and *FN* stands for the false negative, which means that the detected result is a non-fall but it is actually a fall event of the human being. In the case of binary classification, 100% precision score signifies that every element of the positive class verily belongs to the positive class, which is calculated by the equation [33]:

(8) Precision=TP/(TP+FP).

Sensitivity gives the probability of positive result of test data through the equation [33]:

(9) Sensitivity=TP/(TP+FN).

The equation [33] to calculate specificity is given below which provides the probability of negative result of test data.

(10) Specificity=TN/(TN+FP).

F1-score implies the harmonic mean between precision and sensitivity. The following equation, Ref. [33] is used to calculate the F1-score of the test data.

(11) F1−score=(2∗Precision∗Sensitivity)/(Precision+Sensitivity).

### 4.3. Results and Discussion

**Figure 6:**
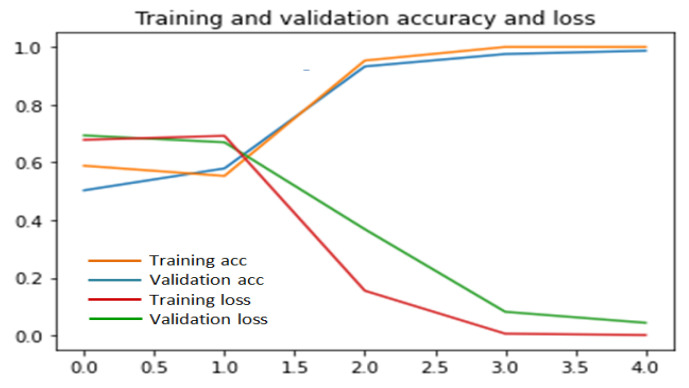
Performance of scratch model for 35% validation and 25% test data.

**Figure 7:**
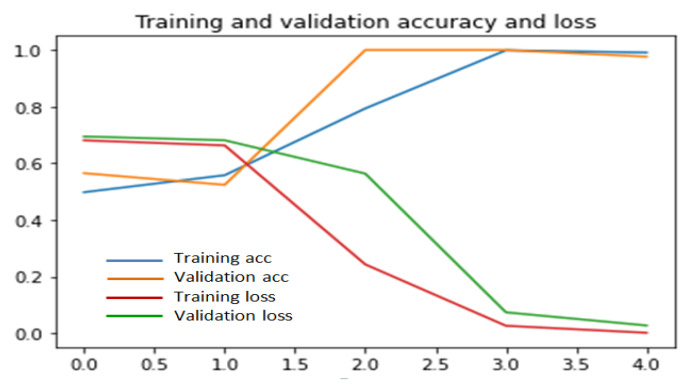
Performance of scratch model for 50% validation and 20% test data.

**Figure 8:**
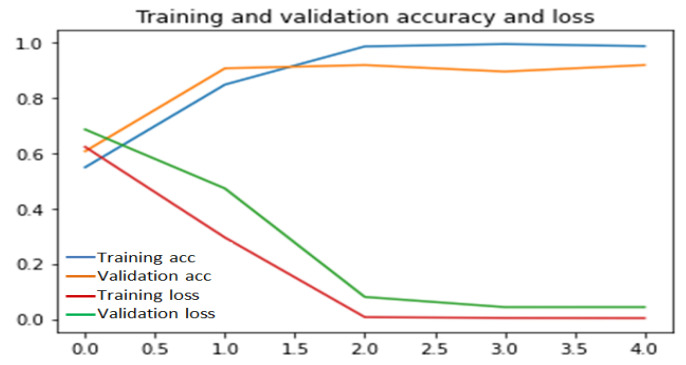
Performance of scratch model for 60% validation and 20% test data.

To implement this scratch model, we have made several experiments on the percentage of training and validation datasets to achieve better accuracy and we have got a mean accuracy of 99% where 99.8% and 98% accuracy for the UR fall detection dataset and Multiple cameras fall dataset, respectively. Multiple cameras fall dataset give less accuracy than the UR fall detection dataset because of the large number of videos in multiple cameras fall dataset. In multiple cameras fall dataset, our model misinterprets when a person lies down intentionally. Our proposed model outperforms when validation data is below 50%, which is shown in Figure 6, Figure 7 and Figure 8. Considering this, 35% videos are used for validation and 25% are used for testing. The rest of the data are used for training.

Figure 6, Figure 7 and Figure 8 illustrate that at 40th epoch, the model achieves the best training and validation accuracy for 40% training, 35% validation and 25% testing data.

**Figure 9:**
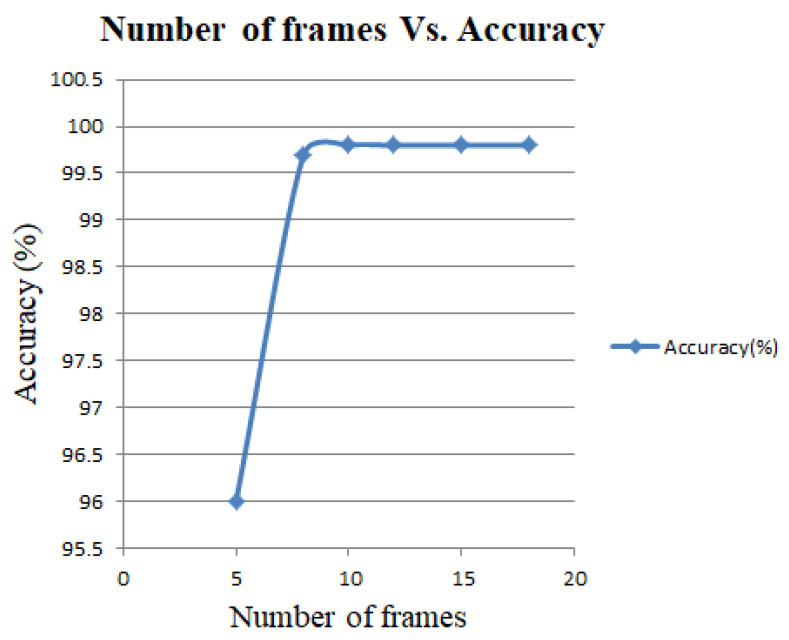
Number of frames vs. validation accuracy curve.

An experiment is done using different frame numbers illustrated in Figure 9. This reveals that fewer than eight frames per video decrease the accuracy rate because it cannot consider all significant frames. Similarly, more than 18 frames per video decrease processing speed because of the large number of computations. The changes of the execution time of the proposed model according to the number of frames in input can be described using Table 2. Here, we see that the fewer the number of frames in input, the faster the execution of the model. However, it decreases the accuracy rate because of the absence of enough information according to Figure 9.

**Figure 10:**
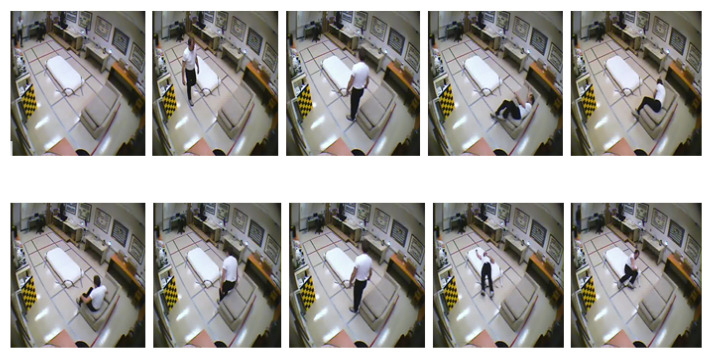
Sequential video frames for daily activity in multiple cameras fall dataset.

**Figure 11:**
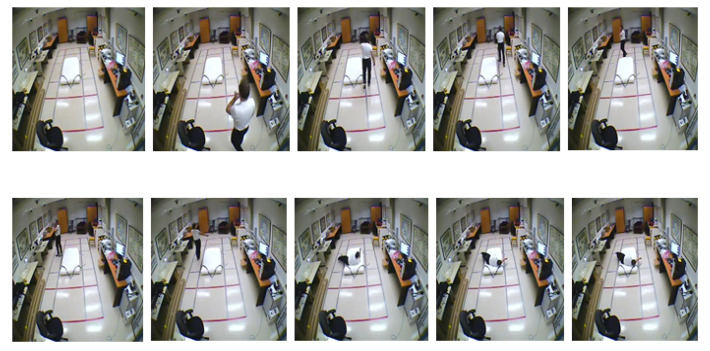
Sequential video frames for fall event in multiple cameras fall dataset.

Therefore we have chosen 10 numbers of distributed frames from the whole video to reduce computational complexity along with better accuracy throughout the whole experiment. Figure 10 and Figure 11 illustrate this 10 numbers of frame sequences of an input video of daily activities and fall events respectively in multiple cameras fall dataset.

As stated earlier, in this scratch model we have performed batch normalization to achieve faster convergence during training. Therefore, Table 3 evaluates the performance using normalized and unnormalized data. It depicts that normalized data achieve a high accuracy rate within a lower number of epochs than unnormalized data.

**Figure 12:**
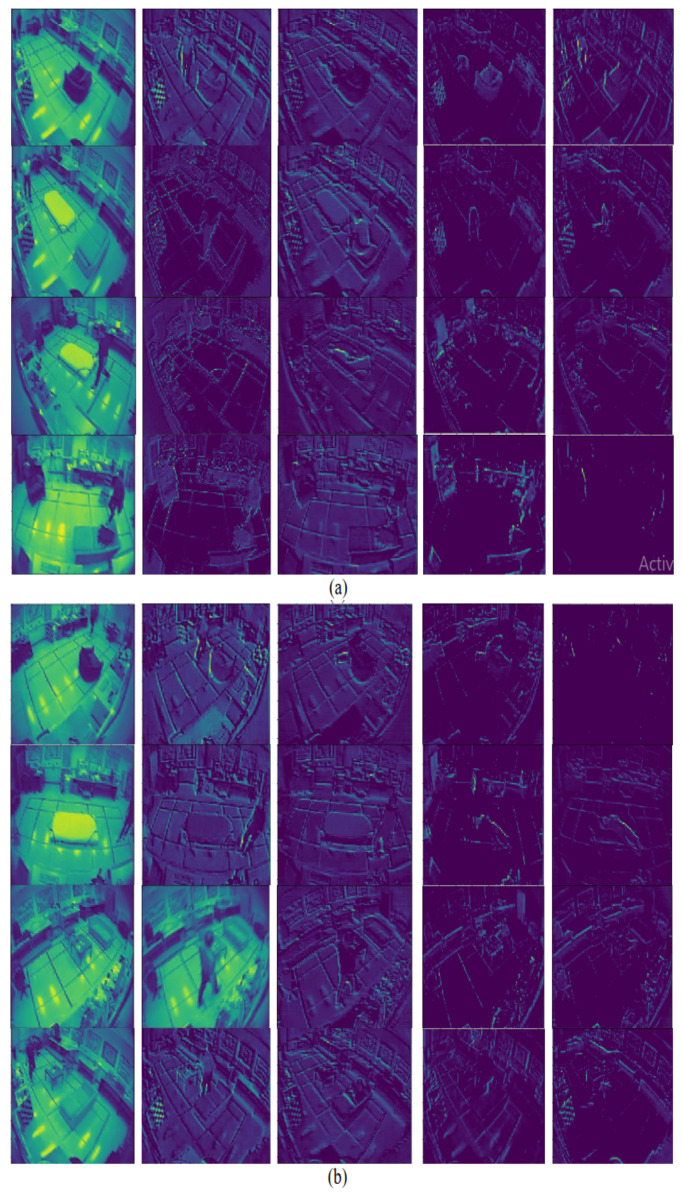
Each row showing low-to-high level feature maps using the proposed convolutional neural network (CNN) model in multiple cameras fall dataset for (**a**) daily activities, (**b**) fall events.

Output of different layers in 5 distributed frames among 10 frames are illustrated in Figure 12. Low to high level feature maps in Figure 12 shows that a deep neural network acts like a black-box, which extracts features of the input image.

At the preliminary stage, the layers extract shallow features like colors and lines while the deep level layers extract the more details patterns. Higher level features are encoded that are difficult to extract and are presented to human readable format [34]. Therefore, deep learning models can extract higher level features that are mystical and more immense in amount than the features considered by humans.

**Figure 13:**
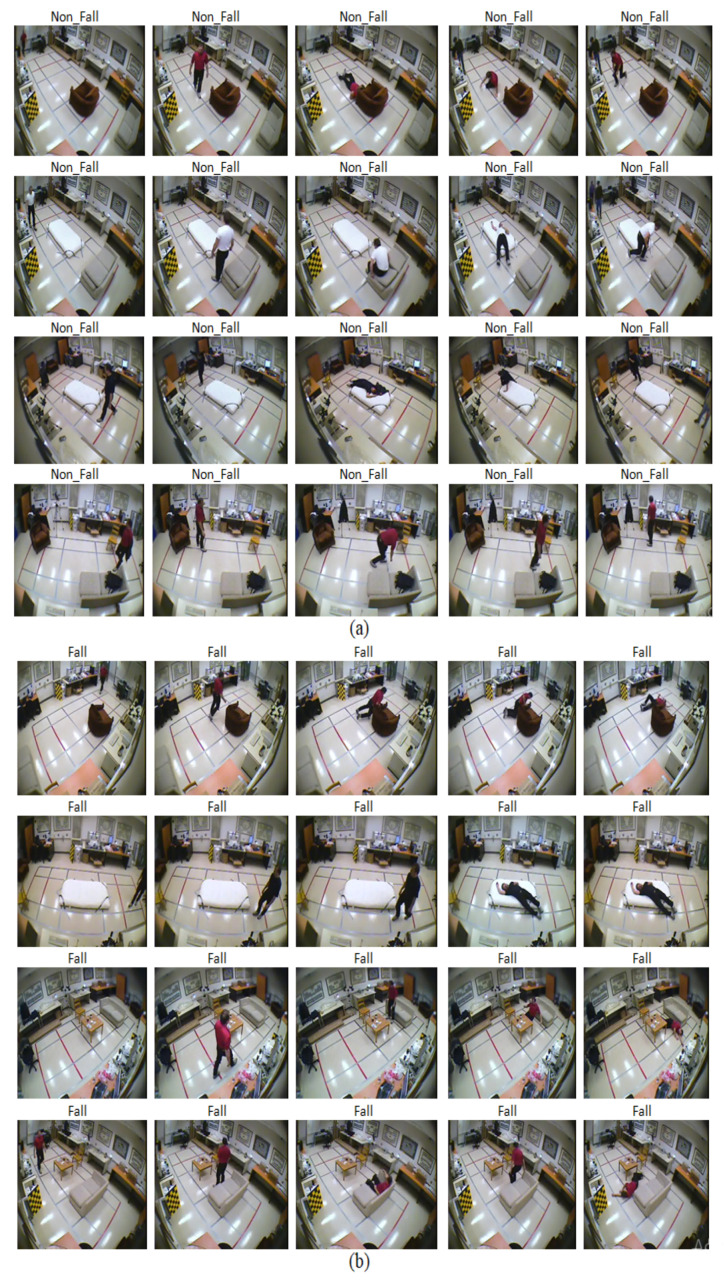
Output of the proposed model for multiple cameras fall dataset for (**a**) non-fall events, (**b**) fall events.

**Figure 14:**
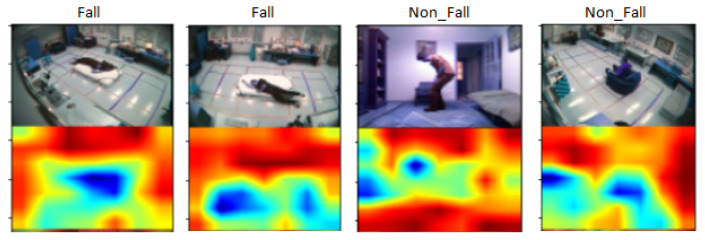
Attention map for fall classification.

Figure 13 shows the output of this experiment to classify human fall events. Here, the first 4 rows represent 5 frames of 4 non-fall event’s videos and the rest of the rows represent 5 frames of 4 fall event’s videos. Figure 14 demonstrates the attention map where the blue region depicts the region of interest after executing this model, which classifies fall and non-fall events.

**Figure 15:**
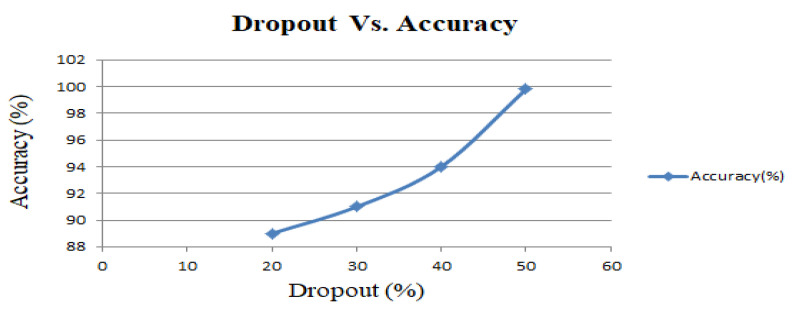
Dropout vs. validation accuracy curve.

Here, a dropout rate of 50% is used in dense layers because more or less than 50% dropout rate gives lower accuracy along with the overfitting problems of training data. The impact of the dropout rate for the change in accuracy is illustrated in Figure 15. This figure illustrates that the proposed model achieves a maximum accuracy of 99.8% at the dropout rate of 50% for the UR fall detection dataset.

Table 4 and Table 5 represent a summary of test accuracy for different existing models where VGG 16 and VGG 19 give 98% test accuracy, Xception generates 99% accuracy but it uses a huge number of parameters compared with our proposed model. As VGG 16, VGG 19, and Xception are pre-trained models, so the number of parameters and depth of these models are observed from [35] where the rest of the others are known experimentally. In [36], the authors classify human fall action using 3DCNN combining with LSTM and obtains an accuracy of 99%. However, 3DCNN comprised with LSTM needs a huge number of parameters. Moreover, training iteration for 2DCNN needs 0.5 s per pass where 3DCNN lasts for 3 s. We have also conducted an experiment using 2DCNN with LSTM and it gives an accuracy of 89% for the same dataset.

It is difficult to estimate the training time of parameters of models as it depends on the GPU model. Using our hardware configuration mentioned earlier and GPU, we have executed some deep learning approaches. Number of parameters, depth, and the training times for these models are represented in Table 6. From this table, it is seen that the training time of our proposed model is faster because of its fewer parameters than others.

**Figure 16:**
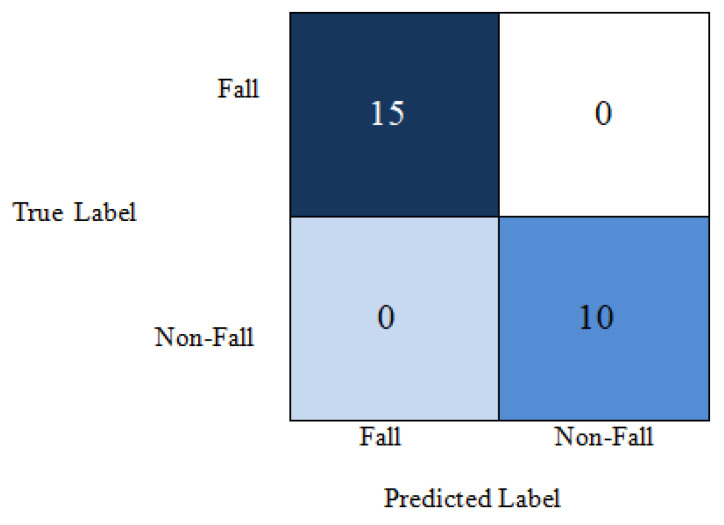
Confusion matrix for UR fall detection dataset.

**Figure 17:**
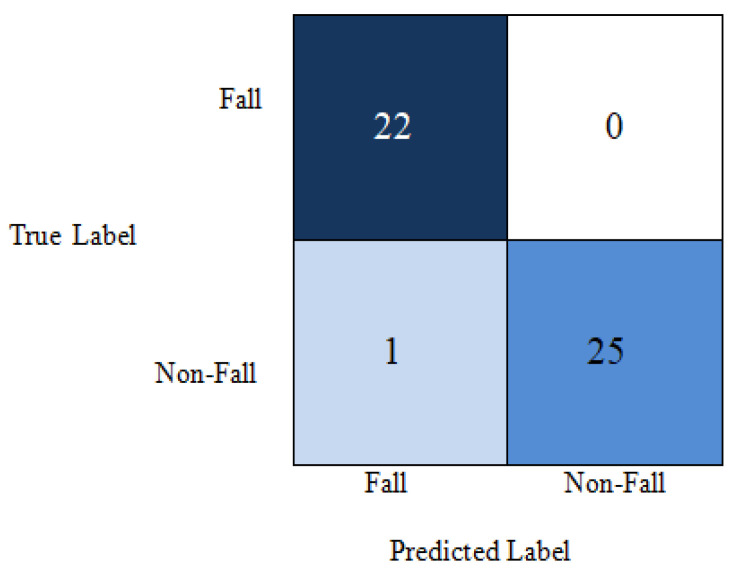
Confusion matrix for Multiple cameras fall dataset.

Considering all these issues, our proposed model gives an average of 99% accuracy using a lower number of parameters along with depth. This is illustrated by the confusion matrix in Figure 16 and Figure 17 for the different datasets. The elements in dark blue diagonal demonstrate the number of correctly classified Fall and Non-Fall events. Here, we see the global accuracy is 100%, which is the ratio of the summation of elements in diagonal by the summation of all elements in the entire matrix. From the confusion matrix for UR fall detection dataset in Figure 16, we see that there are 25 test data points and all are classified correctly. In the confusion matrix for multiple cameras fall dataset in Figure 17, there are 48 test data points. Among them, one non-fall video is misclassified as a fall event.

The class-wise performance like accuracy rate, precision score, sensitivity, specificity, and F1-score of our proposed 2DCNN-GRU model using the UR fall detection dataset and the multiple cameras fall dataset is shown in Table 7 and Table 8.

Table 9 and Table 10 illustrate the comparison of the performance of our proposed model with some existing models. Using the UR fall detection dataset, our model achieves a maximum accuracy of 99.8% compared with Kasturi et al. [17] and Lu et al. [36], which are based on support vector machine (SVM) and 3DCNN followed by LSTM, respectively. On the multiple cameras fall dataset, the proposed model outperforms, acquiring 98% accuracy compared to Wang et al. [26], which is based on PCANet and it is also much higher than Ma et al. [27] based on extreme learning machine (ELM).

## 5. Conclusions

As the deep learning algorithm outperforms other feature extraction algorithms, in this paper, we have proposed a combined architecture where CNN is incorporated with GRU. This is quite challenging to identify human falls at the right time, not only to minimize the negative consequences of a fall but also to increase acceptance level among elderly people. We have used two existing benchmark datasets of fall classification—UR fall detection dataset and multiple cameras fall dataset of variable length videos. The frame number from each video is chosen empirically. To perform classification on these datasets, a scratch model is proposed where CNN followed by GRU is performed, which is our key contribution. Another novelty of our work is we have conducted some transfer learning models along with other deep learning models using the same datasets. However, the proposed model outperforms with an accuracy of 99% with a lower number of parameters than other existing architecture by parameter tunings. Binary cross-entropy loss function outperforms others like mean squared error, hinge loss, and so forth. Despite these, this experiment would be much better if we could enrich our dataset. In the future, we can include an alarm system to take necessary action in time and reduce the after-fall effects. Moreover, the proposed architecture extracts features from the entire frame. Besides, we can reduce the computation time if we can consider only the salient region. Furthermore, this model can also collaborate with people counting techniques to identify the actions of different people residing in the same room.

## Figures and Tables

**Table 1:** Summarization of literature discussion.

Research Paper	Proposed Models	Limitations	Accuracy (%)
[17]	Frame differencing for foreground extraction, ellipse fit for human silhouettes extraction and SVM classifier for fall classification.	SVM underperforms in case of an insufficient training data sample.	96.34%
[18]	Background subtraction method to extract foreground and CNN to classify fall events.	Generates false predictions in bending, crawling, and sitting positions.	90.2%
[20]	CNN for feature extraction and DB-LSTM recognizes sequential pattern.	Shows false projection at the identical background and occluded environment.	92.66%
[21]	CNN to extract the skeleton joint map.	The result can be developed if the recurrent neural network is incorporated.	94%
[23]	Human silhouettes are converted to voxel person and linguistic summarizations of temporal fuzzy inference curves classify fall events.	Not applicable for short-term activity recognition.	96.5%
[24]	Deeper-cut method extracts 2D skeleton and LSTM identify fall actions.	Low accuracy rate.	90%
[25]	Faster R-CNN for fall classification.	Can not properly classify fall events when a person sitting on a sofa or a chair.	95.5%
[26]	PCANet model is trained followed by SVM classifier.	Low accuracy rate.	88.87% (Sensitivity)
[27]	CSS features are extracted from human silhouettes and extreme learning machine (ELM) classify fall events.	Misclassifies the lying position.	86.83%
[28]	OpenPose method extract human skeleton and time-continuous recognition algorithm identify fall event.	Misclassifies workout motions.	97.34%
[29]	OpenPose identify fall events using information from human skeleton.	Unable to classify partially occluded human actions.	97%
[7]	Mask R-CNN extract object from noisy background and Bi-LSTM classify human actions.	Cannot identify the behavior of multiple people living in the same room.	96.7%
[9]	Deep belief network for training and testing human actions.	Not always possible to carry a cell phone in an indoor environment.	97.56% (Sensitivity)
[8]	CNN integrated with LSTM for posture detection.	Unnormalized data may lead to huge training time.	98%

**Table 2:** Effect of number of frames on execution time.

Number of Frames	Execution Time of Proposed Model
5	2.8 min
8	3.9 min
10	4.7 min
12	5.4 min
15	6.8 min
18	7.8 min
20	9.4 min
22	11.7 min

**Table 3:** Effects of batch normalization for performance measurement.

	Training Accuracy	Validation Accuracy	Test Accuracy	Total Epochs
Normalized data	100%	99.7%	99%	40
Unnormalized data	94%	84%	81%	55

**Table 4:** Comparison of test accuracy of the proposed model with existing models in the UR fall detection dataset for human fall classification.

Existing Models	Accuracy (%)
VGG 16	98%
VGG 19	98%
Xception	99%
1DCNN with GRU	94.30%
2DCNN with Bi-LSTM	95.50%
3DCNN with LSTM	99%
2DCNN with LSTM	89%
Proposed Model	99.80%

**Table 5:** Comparison of test accuracy of the proposed model with existing models in multiple cameras fall dataset for human fall classification.

Existing Models	Accuracy (%)
VGG 16	97.60%
VGG 19	98%
Xception	98%
1DCNN with GRU	92.70%
2DCNN with Bi-LSTM	95%
3DCNN with LSTM	97.50%
2DCNN with LSTM	88%
Proposed Model	98%

**Table 6:** Number of parameters, depth and training time comparison with existing models for human fall classification.

Existing Models	Number of Parameters	Depth	Training Time
VGG 16	138,357,544	23	39 m 21 s
VGG 19	143,667,240	26	46 m 31 s
Xception	22,910,480	126	18 m 22 s
3DCNN with LSTM	12,317,230	20	11 m 44 s
2DCNN with LSTM	7,523,320	18	7 m 16 s
Proposed Model	5,288,860	18	4 m 7 s

**Table 7:** Class-wise performance of the scratch model for UR fall detection dataset.

Classes	Mean Accuracy (%)	Accuracy (%)	Precision (%)	Sensitivity (%)	Specificity (%)	F1-Score (%)
Fall event	100	100	100	100	100	100
Non-fall event		100	100	100	100	100

**Table 8:** Class-wise performance of the scratch model for Multiple cameras fall dataset.

Classes	Mean Accuracy (%)	Accuracy (%)	Precision (%)	Sensitivity (%)	Specificity (%)	F1-Score (%)
Fall event	98	100	100	96	100	98
Non-fall event		96.15	100	96	100	98

**Table 9:** Performance comparison with existing models using UR fall detection dataset.

Methods	Accuracy (%)
Kasturi et al. [17]	96.34%
Lu et al. [36]	99.27%
Proposed model	99.8%

**Table 10:** Performance comparison with existing models using multiple cameras fall dataset.

Methods	Accuracy (%)
Wang et al. [26]	96%
Ma et al. [27]	97.2%
Proposed model	98%

## Data Availability

The authors have used publicly archived dataset named UR Fall Detection Dataset and Multiple cameras fall dataset for validating the experiment. The dataset is available at http://fenix.univ.rzeszow.pl/~mkepski/ds/uf.html (accessed on 18 February 2021) and http://www.iro.umontreal.ca/~labimage/Dataset/ (accessed on 18 February 2021).

## Abbreviations

The following abbreviations are used in this manuscript:

CNN	Convolutional Neural Network
RNN	Recurrent Neural Network
GRU	Gated Recurrent Unit
ReLU	Rectified Linear Unit
DB-LSTM	Deep Bi-directinal Long Short Term Memory
